# From Sweetness to Mouthfeel: A Review on Overcoming Sensory Barriers in Sugar‐Free Beverages

**DOI:** 10.1155/ijfo/2345722

**Published:** 2025-11-22

**Authors:** Imogen Ramsey, Jing Liu, Jan Hendrik Swiegers, Olayide Oladokun

**Affiliations:** ^1^ Carlsberg Research Laboratory, Carlsberg Group, Copenhagen, Denmark; ^2^ Carlsberg Group Development, Carlsberg Group, Copenhagen, Denmark

**Keywords:** artificial sweeteners, flavours with modifying properties (FMPs), mouthfeel enhancer, natural sweeteners, positive allosteric modulators (PAMs), temporal profile

## Abstract

**Background:**

Over recent decades, considerable research has focused on understanding sweet taste mechanisms and developing new noncarbohydrate sweeteners due to rising noncommunicable diseases. New regulations limiting high sugar levels in food and beverages have driven innovation towards reduced‐sugar and sugar‐free products, often using noncarbohydrate sweeteners. Extensive research was aimed at improving their physical and sensorial properties. However, to the authors′ knowledge no publication has taken a holistic approach by integrating all major sensory and functional challenges of sugar reduction in beverages in one structured framework.

**Scope and Approach:**

This review addressed these challenges, which included (i) sweetness, (ii) temporal profile in relation to sweetness onset and linger, (iii) masking, (iv) flavour intensity and delivery and (v) mouthfeel enhancement. It firstly discussed the strategy of replacing the sweetness of sugar by utilizing different sweeteners, investigating mechanisms behind the sensory characteristics and solutions and exploring artificial and natural sweeteners and positive allosteric modulators for sweetness. For the temporal profile, methods to measure sweetness onset and linger were investigated. Solutions for masking off‐tastes and flavours were discussed, including bitter‐blockers, blending of sweeteners, production technologies and flavours with modifying properties. Changes in flavour intensity and delivery were discussed. In the last section, strategies for addressing mouthfeel reduction were summarised including the usage of hydrocolloids, sugar alcohols, fibre syrups and mineral salts.

**Key Findings and Conclusions:**

Overall, the need for clearer guidelines and fewer restrictions was debated, highlighting conflicting consumer perspectives on the need for healthy, natural and more sustainable products and regulatory barriers around novel solutions. The advancement of scientific knowledge is emphasised, and collaboration between cross‐functional teams is discussed as essential to move sugar‐free products closer to their sugar‐containing counterparts.

**Summary**

• Reducing and replacing sugar within beverages is challenging due to its multifaceted role.
• Noncarbohydrate sweeteners were discussed as strategies to replace sweetness.
• Temporal profiles, including sweetness onset and lingering, were reviewed.
• Strategies to mask sweetener off‐tastes using flavours with modifying properties (FMPs) were discussed.
• Alternatives for replicating the mouthfeel of sugar in beverages were reviewed.

## 1. Introduction

Over the past few decades, the fast‐moving consumer goods (FMCG) industry has been tasked by both the World Health Organization (WHO) and local government policies with reducing sugar within both food and beverage products due to links between high sugar intake and increased health concerns, such as obesity, dental caries and diabetes [1]. Sugar‐sweetened beverages are one of the main culprits, with a recent study showing that millions of cases of diabetes and heart disease are linked to the regular consumption of sugary drinks [2]. Thus, the Union of European Soft Drinks Associations (UNESDA) has also committed to reducing average sugar in products by 10% by 2025 [3]. Yet humans have an innate preference for sweet products, stemming from thousands of years ago, due to an aversion to bitter foods that are often poisonous to humans and a believed evolutionary advantage that sweeter products meant more energy‐dense foods [4].

Regulatory practices and new product innovations have been used to try to accelerate reaching these targets in sugar reduction. Regulatory practices include the implementation of sugar taxes, which increase the price of targeted products for consumers, making products less accessible to consumers, reduction of portion sizes, educational interventions and advertising regulations [5–10]. From a business perspective, cost‐saving strategies through the reduction of high‐cost commodities, such as sucrose, by either reduction or replacement with high‐intensity sweeteners have contributed to change. In addition to this, internal commitments have also increased focus, with the Coca‐Cola Company committing that by 2025, 50% of their drinks portfolio in Europe will be low‐ or no‐calorie [11]. Another suggestion is the idea of a stepwise procedure to reduce consumer perception of sweetness over a period of years. In a recent study in the United States and Mexico, which used both a stepwise and direct decrease of sweetness procedure, a decrease in sweetness intensity ratings for low‐calorie sweetened beverage consumers over 6 months was found [12]. It was discussed that this could be due to gradual desensitisation and sensory acclimatisation, with sweet taste receptors able to adapt slowly, leading to decreased sensitivity to sweetness [13]. However, for this strategy to work, all food and beverage companies need to be onboard with such a change (something that can only really occur if there is a government objective in place).

New product innovations to meet the consumers′ demands for lower sugar intake have been developed by food and beverage manufacturers whilst also preserving the desirable sensory characteristics. For many years this focus meant that artificial sweeteners such as aspartame, acesulfame K (Ace‐K), saccharin and sucralose were the market leaders in the beverage sector due to their similar (although not perfect) sweetness profile compared to their sucrose counterpart. Nonetheless, in June 2023, the WHO cancer research department declared the artificial sweetener aspartame as ‘possibly carcinogenic to humans’, causing an upheaval for many food and drink businesses using this artificial sweetener in their products [14]. At a similar time, WHO also advised not to use nonsugar sweeteners for weight control or to reduce the risk of noncommunicable diseases. This was due to findings from a systematic review showing no long‐term benefit in reducing body fat and potential undesirable effects on the risk of Type 2 diabetes, cardiovascular diseases and mortality [15]. Consequently, there is growing evidence that some consumers are now rejecting artificially sweetened products for more natural propositions, with the natural sweetener market predicted to be worth $39 billion revenue by 2026 [16, 17], thus presenting an opportunity for companies producing natural sweeteners such as steviol glycosides and monk fruit.

Considerable research has been conducted to understand consumer perceptions on sugar reduction, as well as on the healthiness of sugar alternatives [18–21]. In 2022, EFSA conducted social research with 7469 citizens across EU member states. When asked about food choice and eating behaviours, unsurprisingly taste was the most important factor, followed by price and then health. In addition, 49% of consumers chose an alternative reduced‐sugar product or one containing sugar substitutes (such as artificial sweeteners) to regular products [19]. In multiple studies, consumers preferred stevia, monk fruit and raw sugar over high fructose corn syrup, aspartame and sucralose, with many seeing these as healthier options, leading the authors to conclude that consumers link healthiness to a sweetener′s level of naturalness [20, 21].

Unfortunately, sugar reduction and replacement are not as simple as one might think. There are numerous ways in which sugar is important within beverages, which include physical and chemical characteristics. Sugars have been found to suppress bitterness, saltiness and sourness through chemical, oral physiological and cognitive interactions [22, 23]. They can enhance flavour intensity and delivery [24, 25] and bring higher viscosity in solutions, resulting in a thicker and fuller mouthfeel [26, 27]. Finally, it is an important ingredient for preservation [28]. With one ingredient contributing all these key properties, there are clearly challenges when it comes to sugar reduction and removal in beverages. The industry has mainly focused attention on calorie reduction using high‐intensity sweeteners, yet these products come with increased sweetness linger and off‐flavours, problems with stability over time and decreased flavour delivery and are therefore not able to deliver the expected sensorial properties that consumers expect compared to sugar‐containing counterparts. In recent years, research has focused on reviewing and improving high‐intensity natural sweeteners due to increased interest in natural and ‘clean‐label’ products [29–33]; however, to the authors′ knowledge, no publication has taken a holistic view by integrating all the major sensory and functional challenges of sugar reduction (sweetness, temporal profile, masking, flavour delivery and mouthfeel) in one structured framework. This positions the review as the first comprehensive map of how sugar impacts multisensory perception and how each element can be strategically replaced or mimicked.

**Figure 1:**
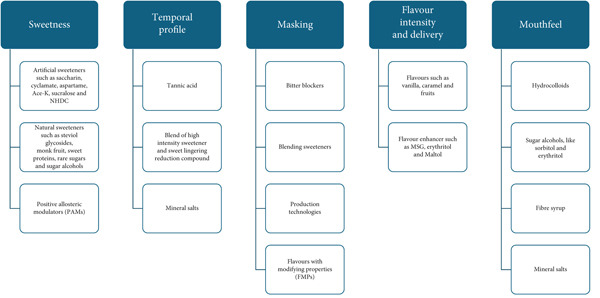
Overview of the holistic approach in the product development process of sensory challenges that occur by removing or replacing sucrose. These include sweetness, temporal profile, masking, flavour intensity and delivery and mouthfeel (shown in blue). Suggested recommendations to tackle each challenge are shown in white.

In the first section of the review paper, strategies to tackle the replacement of sweetness due to the removal of sugar will be discussed through a brief overview of the mechanism of sweet taste, and solutions to counteract this through artificial and natural sweeteners, as well as positive allosteric modulators (PAMs). Secondly, the challenges faced in trying to emulate the unique temporal profile of sugar will be discussed in relation to sweetness onset and linger. Next, the mechanism for sugar masking of off‐tastes will be reviewed, with strategies such as bitter blockers, blending of sweeteners and the inclusion of FMPs. Then, flavour intensity and delivery including flavour interaction and suppression/enhancement will be summarised. Finally, mouthfeel enhancement strategies will be reviewed. These properties, as well as their proposed solutions, are all summarised in Figure 1.

## 2. Sweetness

The main sensory property that sugars are well known for is their sweetness. Of course, when removing sugars from a product, then a solution is needed to help deliver a similar sweetness intensity. The main principle to solve this challenge is by looking into molecules which can interact with the sweet receptor in a similar way to sucrose, with these molecules divided into artificial and natural sweeteners. A sweetener is a food additive which mimics the taste and effect of sugar. For the purposes of this review, artificial sweeteners can be described as synthetic substances, meaning not found in nature, with natural sweeteners described as all sweeteners derived from a natural source (this does not include natural products such as honey, agave, molasses, coconut sugar and date sugar). These will be discussed in more detail with an introduction to the sweetener, physical, chemical and sensory properties, as well as regulatory and labelling information. For new ingredients to be used in food stuffs, such as novel sweeteners, extensive research and approval are needed by regulatory agencies. This includes complete chemical characterisation of the ingredient, studies on functionality and stability, method of manufacture, detection method with data validating the analytical method and toxicological research. A summary of all sweeteners and their properties, as well as advantages and disadvantages, is shown in Table 1. Regulatory limits and labelling for different beverage types within the EU are further shown in Table 2.

**Table 1:** Advantages and disadvantages of artificial and natural sweeteners, with a focus on sweetness intensity; physical, chemical and sensory properties; regulatory and labelling and health and environmental concerns.

**Type**	**Sweetener**	**Sweetness equivalence to sucrose**	**Advantages**	**Disadvantages**
Artificial	Saccharin	200–700x	• Physical properties: Readily water‐soluble, relatively stable [34]	• Sensory properties: Bitter/metallic aftertaste, sweetness linger [35, 36] • Regulatory: Banned by FDA in 1997 due to link between consumption and bladder cancer in rats. Further research later disproved this claim; however, the reputation still remains. • Environmental concerns: Not readily biodegradable in the environment, but little concern in terms of toxicity at current environmental concentrations
	Cyclamate	30–50x	• Physical properties: Both forms are fully water‐soluble • Sensory properties: Similar temporal profile to that of sucrose	• Sensory properties: At high concentrations can have bitter and salty tastes • Health: Certain members of population (20%) can metabolise to form cyclohexylamine, which can be toxic • Regulatory: Banned by FDA in 1969 due to link between consumption of cyclamate: Saccharin mixture and bladder cancer. Further research again disproved this claim, but reputation still remains. • Regulatory: Not allowed for use in beer, malt‐based beverages or cider. • Environmental concerns: Cyclamates being found in water bodies, with research suggesting plant cytotoxic and mutagenic effects at permitted levels
	Aspartame and derivatives	200x	• Sensory: Flavour enhancing properties	• Physical: Degrades in solution and only stable at pH 3–5, with sweetness lost during heat treatment [37] • Sensory: Sweetness onset takes longer to appear and lingers longer [37] • Environmental concerns: At high concentrations, it has been found to be hazardous to aquatic environments, yet there is no risk at the concentrations found in nature currently [38]
	Acesulfame K	200x	• Physical properties: Highly soluble in aqueous solutions and at high temperatures, with no reactions with food constituents observed [37]	• Sensory properties: Slight bitter aftertaste • Environmental concerns: Not metabolised by the human body, therefore able to enter wider environment [38]
	Sucralose	600x	• Physical properties: Soluble in water, ethanol, methanol and maltodextrin and stable over a wide range of pH and temperatures [37] • Sensory properties: Time intensity sweetness profile similar to sucrose	• Health: Some reports of causing migraines and changes in gut microbiota • Environmental concerns: Not degraded by wastewater treatment processes and therefore has been found to be accumulating in effluents, marine and coastal waters, surface waters and drinking water. Minimal concerns at the concentrations present in the environment; however, in the future, it could be seen as a contaminant, especially for microenvironments [38]
	Neohesperidin dihydrochalcone	1900x	• Health: Antioxidative, anti‐inflammatory and antiapoptotic properties have been reported [39]	• Sensory properties: Liquorice taste that tends to linger • Regulatory: Not approved for use as a food additive in the United States, Canada and Australia
Natural	Steviol glycosides	300x	• Sweetness intensity: Around 300 times sweeter than sucrose • Physical properties: Stable between pH 4 and 8 • Environmental concerns: Precision fermentation of steviol glycosides shows potential for having lowest LCA score [40]	• Sensory properties: Liquorice, bitter and metallic aftertastes • Physical properties: Reb M and Reb D have been found to be insoluble in water
	Monk fruit	250x	• Sweetness intensity: Around 100–300 times sweeter than sucrose • Health: Research suggests mogrosides contain bioactive properties	• Sensory properties: Characterised by a lower peak sweetness, additional off‐tastes (bitter, chemical and metallic) and longer lasting sweetness when compared to sucrose [41, 42] • Regulatory: Not approved for use as food ingredient in the EU
	Sweet proteins	Thaumatin: 1600–3000x Brazzein: 2000x	• Physical properties: Many unstable at temperatures above 50°C [43]; however, thaumatin and brazzein are more heat stable. Thaumatin is soluble in water and low levels of alcohol and stable at different pH levels and up to 120°C. Brazzein is water‐soluble, stable between pH 2.5 and 8 and heat stable up to 80°C. • Sensory properties: Thaumatin has been found to mask bitter and astringent notes, thus being used as a flavour modifier. Brazzein has shorter aftertaste than other sweeteners on the market and can enhance flavour in beverages with citric acid [44] • Health: Brazzein has more recently been found to have anti‐inflammatory, antiallergic and antioxidant effects [45]	• Sensory properties: Thaumatin has delayed onset of sweetness and long‐lasting lingering effect [37] • Regulatory: Thaumatin only protein approved for use in the United States and the EU. Brazzein only approved for use in the United States • Health: Some discussion on potential of sweet proteins being allergens due to their structure and sequence similarities to other allergens, but no direct correlation shown
	Rare sugars	D‐allulose, D‐sorbose and D‐allose: 0.7–0.8x D‐tagatose: 0.92x	• Sensory properties: D‐allulose has similar sweetness decay to sucrose [42]. At concentrations of 4.5%–18%, D‐tagatose has no undesirable off‐tastes • Physical properties: D‐allulose has high solubility	• Sensory properties: At high concentrations, D‐tagatose has a slight chemical‐like side taste • Regulatory: D‐allulose and D‐tagatose only have GRAS status
	Sugar alcohols	Erythritol: 0.6–0.8x	• Physical properties: Erythritol highly soluble and stable at high temperature and pH [34] • Sensory properties: Can improve mouthfeel and mask unwanted off‐flavours [37] • Health: Recent research has shown that polyols have good antioxidant properties, reducing stress responses, as well as prebiotic properties which can contribute to healthy intestinal microbiota [46]	• Health concerns: Excessive consumption can lead to gastrointestinal symptoms [47], and therefore products with more than 10% need an advisory statement • Regulatory: At low levels (1.6%), erythritol can be used in nonalcoholic flavoured beverages but cannot be used in alcoholic drinks in the EU

**Table 2:** Regulation and labelling standards for artificial and natural sweeteners within the EU.

**Type**	**Sweetener**	**E-number**	**Acceptable daily intake (ADI) (mg/kg body weight)**	**Maximum limit**			
				**Flavoured drinks (energy reduced or no added sugar) (mg/L)**	**Beer and malt beverages (including alcohol-free counterparts (mg/L)**	**Cider (including alcohol-free counterparts (mg/L)**	**Other alcoholic drinks (including mixtures of alcoholic drinks with nonalcoholic drinks and spirits with less than 15% of alcohol) (mg/L)**
Artificial	Saccharin	E954	0–5	80 (100 in ‘gaseosa’)	Below 1.2% ABV: 80	80	80
	Cyclamate	E952	0–7	250	N/A	N/A	250
	Aspartame	E951	0–40	600	Below 1.2% ABV: 600 Energy reduced beer: 25	600	600
	Neotame	E961	0–2	20 (2 as flavour enhancer)	Below 1.2% ABV: 20 Energy reduced beer: 1	20	20
	Advantame	E969	0–5	6	Below 1.2% ABV: 6 Energy reduced beer: 0.5	6	6
	Salt of aspartame–acesulfame	E962	—	350	Below 1.2% ABV: 350 Energy reduced beer: 25	350	350
	Acesulfame K	E950	0–15	350	Below 1.2% ABV: 350 Energy reduced beer: 25	350	350
	Sucralose	E955	0–15	300	Below 1.2% ABV: 250 Energy reduced beer: 10	50	250
	Neohesperidin dihydrochalcone	E959	0–5	30 (50 in milk and milk derivatives)	Below 1.2% ABV: 10	20	30

Natural	Steviol glycosides	E960a–960d	0–4	80	Below 1.2% ABV: 70	N/A	150
	Monk fruit (nonselective water extract)	N/A	N/A	N/A	N/A	N/A	N/A
	Sweet proteins: Thaumatin	E957	N/A	0.5 (only water‐based flavoured nonalcoholic drinks, as flavour enhancers only)	N/A	N/A	N/A
	Rare sugars	N/A	N/A	N/A	N/A	N/A	N/A
	Sugar alcohols: Erythritol	E968	0.5	16,000 (as flavour enhancer only)	N/A	N/A	N/A

### 2.1. Mechanism

**Figure 2:**
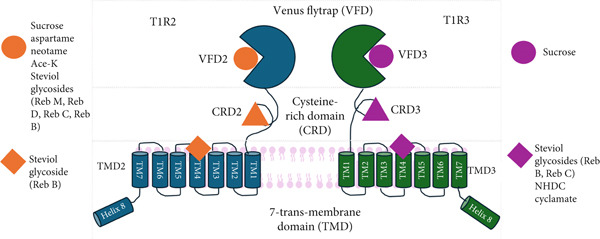
Schematic of human heterodimeric sweet taste receptor (adapted from [54]). The T1R2/T1R3 receptor features multiple binding sites for sweet ligands. Aspartame, neotame, acesulfame K (Ace‐K), rebaudioside M (Reb M), rebaudioside D (Reb D), rebaudioside C (Reb C) and rebaudioside B (Reb B) preferentially bind to the VFD2 domain. Sucrose binds to both the VFD2 and VFD3 domains. The TMDs engage with various allosteric modulators and sweet compounds. Sweeteners like Reb B bind to TMD2. Additionally, Reb B, Reb C, neohesperidin dihydrochalcone (NHDC) and cyclamate bind to TMD3.

Research has been conducted over many years to elucidate the mechanism behind sweet taste. Sweet taste is first sensed through the taste buds, located on papillae all over the tongue, epiglottis and palate [48, 49]. Sweet compounds also stimulate the gastrointestinal system, brain and other organs via gustatory mechanisms. Before extensive research was carried out into the discovery of the sweet taste receptor, it was believed that the five basic taste modalities (sweet, sour, bitter, salty and umami) were sensed in certain areas of the tongue [50]. However, later, this research was disproved, and it was shown that each taste modality can be sensed on every part of the tongue at different detection thresholds [51]. It has now been widely reported that the cells responsible for sweet taste have specialised proteins, called G protein‐coupled receptors (GPCRs), that function as receptors for sweet tastants. These two subunits, called T1R2 and T1R3, have a venus flytrap mechanism, which means that when open, a sweetener can bind to the exposed pocket. Sucrose binds to both venus fly trap domains of the T1R2/T1R3 receptor. This then triggers the venus fly trap to close and activates the receptor by stabilising the closed conformation, thus sending a signal to the brain that is interpreted as a sweet taste [52]. Other sweeteners, such as sucralose and aspartame, have been found to bind to only one specific domain of T1R2 [53], whilst steviol rebaudiosides bind to four different sites of T1R2 and T1R3 [54], thus, explaining some of the differences between the sweetness quality of noncarbohydrate sweeteners in comparison to sucrose. Figure 2 (adapted from Hao et al. [54]) has been added to increase clarification in this paper.

### 2.2. Artificial Sweeteners

Numerous artificial sweeteners have been discovered over recent centuries, which include saccharin, cyclamate, aspartame and derivatives, Ace‐K, sucralose and neohesperidin dihydrochalcone (NHDC). Here, they will be briefly discussed in terms of history, physical, chemical and sensory properties and health; however, more detailed reviews are available [34, 37].

#### 2.2.1. Saccharin

Saccharin is the oldest artificial sweetener, discovered in 1879 by Constantine Fahlberg at Johns Hopkins University in the United States. It is a noncarbohydrate sweetener of 1,2‐Benzisothiazol‐3(2H)‐one and formed by electrochemical oxidation with the help of potassium permanganate and chromic acid. It is marketed under the brand names Sweet′N Low, Sugar Twin and Necta Sweet. As the acid form of saccharin is sparingly soluble in water, the sweetener is usually used as the sodium or calcium salt. Both salts are readily water‐soluble, with a solubility of 100% at 20°C for sodium saccharin and 37% at 20°C for calcium saccharin. Stability is not affected by temperature and pH normally encountered in manufacturing [34]. Saccharin is around 200–700 times sweeter than sucrose, yet one of the most noted drawbacks is its bitter or metallic aftertaste and lingering sweetness [35, 36]. Saccharin is permitted for use in more than 100 countries, after being declared safe for human consumption.

#### 2.2.2. Cyclamate

The sweetening properties of cyclohexyl sulfamic acid, or cyclamate, were discovered by researchers at the University of Illinois [55]. It is commonly used as a sweetener either in its sodium or calcium salt forms, which are fully soluble in water. Cyclamate is around 30 times sweeter than sucrose, with the lowest sweetening power of the artificial sweeteners, yet it has been found to have a similar temporal profile to that of sucrose. At high concentrations, a bitter and salty aftertaste has been observed [37]; however, due to its low sweetening power, it is often used in blends, usually with saccharin at a 10:1 ratio that has been shown to have a good synergistic effect [56]. Cyclamates are approved for use in more than 50 countries; however, they are not approved for use in the United States, Japan, Mexico and South Korea. This is because although cyclamate itself has very low toxicity, during consumption, it can be metabolised by gut bacteria to form cyclohexylamine, which has been shown to be toxic. Interestingly, there are large interindividual variations in metabolization rates, meaning different levels of cyclohexylamine can be formed amongst individuals, with some known as nonconverters (around 80% of the population) and others known as converters [57]. In 1969, cyclamate was banned by the FDA due to suspicions of links to bladder cancer, following a study where rats were fed a 10:1 cyclamate–saccharin mixture. Other toxicological and carcinogenic studies were conducted later using the same cyclamate–saccharin solutions and breakdown product cyclohexylamine, with these showing a negative carcinogenic effect.

#### 2.2.3. Aspartame and Derivatives

Aspartame was discovered entirely by accident during tests to find antiulcer drugs, in which scientist James Schlatter licked his finger during an experiment and found it to be sweet [58]. It is one of the most widely used and consumed sweeteners today, with the structure consisting of a methyl ester of the dipeptide of the amino acids aspartic acid and phenylalanine. Trademark names include Equal, Nutrasweet and Candere [59]. In terms of sensory properties, aspartame is around 200 times sweeter than sucrose. Unfortunately, the sweetness profile is not a direct match to sucrose, as the onset takes longer to appear and lingers longer [37]. Derivatives of aspartame are also available, namely, advantame and neotame; however, both are rarely used. Advantame is around 100 times sweeter than aspartame, does not have a bitter aftertaste and is stable at low pH and high temperatures [37]. Neotame also increases apparent sweetness to approximately 7000–13,000 times that of sucrose [60]. Aspartame is soluble in water at approximately 1% at 25°C, with increased solubility attained at higher temperatures [34]. However, it is well known to degrade in solution, with some sweetness lost during heat treatment, with the most optimal range of pH stability found to be between 3 and 5 [37]. In addition, it can also lose its sweetness after reacting with several flavourings, aldehydes or ketones, but can have some flavour‐enhancing properties, especially with soft fruit flavours [61]. After ingestion, aspartame breaks down into aspartic acid, phenylalanine and methanol, which are all usually absorbable in the intestinal mucosa. However, for some born with the rare inherited disease called phenylketonuria (PKU), these high levels of phenylalanine can be a health hazard; therefore, products with aspartame need to be labelled with a phenylalanine statement [59]. Aspartame has been a source of regular debate between health officials and recently was in the news due to reports by the WHO and the International Agency for Research on Cancer (IARC) classifying it as possibly carcinogenic to humans [62].

#### 2.2.4. Ace‐K

Acesulfame potassium, usually referred to as Ace‐K, is a potassium salt of 6‐methyl‐1,2,3‐oxathiazine‐4(3H)‐one‐2,2‐dioxide, discovered in 1967 by German chemist Karl Clauss. This sweetener is marketed under the brand names Sunett and Sweet One. It is a crystalline solid with good water solubility (27% at 20°C), is stable in solid form and adequately stable to hydrolysis, temperature and light exposure for use in beverage applications [34]. Ace‐K is about 200 times sweeter than sucrose but has a slight bitter aftertaste; therefore, it is often used in combination with other sweeteners (aspartame or sucralose) to enhance synergistic effects, with a quick sweetness onset [63, 64]. It has been extensively studied for safety and has been approved for use by various health authorities.

#### 2.2.5. Sucralose

Sucralose is made from sucrose by selective chlorination, which substitutes three chloride atoms for three hydroxyl groups. This new molecule was discovered by Tate and Lyle, PLC in a wider research study exploring the mechanism for the sweet taste of sucrose, with molecules carefully synthesised and evaluated to determine the spatial structure and molecular configuration required for sweetness perception [65]. Sucralose has been marketed under the brand name Splenda, but due to its intense sweetness, it is normally mixed with the bulking agent, maltodextrin, and thus the commercialised product only contains around 1.1% sucralose [66]. Sucralose is soluble in water, ethanol, methanol and maltodextrin and is stable over a wide range of pH and temperatures; therefore, it can be used in a number of applications [37]. It has a pleasant, sweet taste that is around 600 times that of sucrose, and its time intensity (TI) sweetness profile is similar to that of sucrose. In some studies, it has been found to have a bitter off‐taste, but this was explained to be due to the ability of sucralose to activate both sweet and bitter taste receptors (TAS2Rs) [67]. After extensive research on safety and intended technical effects, sucralose was approved for use as a general‐purpose sweetener by the FDA in 1999.

#### 2.2.6. NHDC

The last of the sweeteners permitted in the EU is NHDC. It is a flavone glucoside, and although not present in nature, it is structurally related to flavonoids and their corresponding dihydrochalcones occurring in many plants [68]. Flavonoids present in citrus fruits include naringin (grapefruit) and hesperetin (lemon and sweet oranges). The source material, neohesperidin, is naturally occurring in bitter oranges and is isolated by alcohol extraction [69]. This sweetener is 1900 times sweeter compared to sucrose and is often used in combination with other sweeteners as it has a liquorice sweet aftertaste that tends to linger. NHDC is approved for use as a food additive in the EU, but not in the United States, Canada and Australia. No human studies on the effect of NHDC are available; however, animal and cell studies have shown that it is not toxic. Interestingly, more recent studies have shown antioxidative, anti‐inflammatory and antiapoptotic properties [39].

### 2.3. Natural Sweeteners

Due to increased consumer interest in natural and clean label ingredients, recent research has looked at extracting and improving the sensory properties of sweeteners from natural sources. Here, steviol glycosides, monk fruit, sweet proteins, rare sugars and sugar alcohols will be discussed in terms of history, physical, chemical and sensory properties, production methods and health; however, more detailed reviews are available [29, 30, 32, 34, 37, 70].

#### 2.3.1. Steviol Glycosides

Steviol glycosides are extracted from *Stevia rebaudiana*, a small perennial herb endemic to South America. These glycosides naturally occur in the leaves, with the total content around 4%–20% dependent on genotype and culture conditions. Stevia leaves also contain proteins, carbohydrates, lipids, dietary fibres, oils, vitamins and phenolic compounds [71]. It should also be noted that some steviol glycosides can also be extracted from *Rubus suavissimus* (sweet tea). Currently there are four different methods for the production of steviol glycosides, which include extraction from stevia leaves, bioconversion of this extract into more purified steviol glycoside rebaudiosides D and M, glucosylated steviol glycosides or precision fermentation. There are around 30 different steviol glycosides, but the ones most researched include dulcoside A, rebaudiosides A–E, steviolbioside and stevioside. Different glycosides have the same steviol backbone with a varying number of glucose molecules attached [72]. Stevioside is the major component from stevia leaves (9.1%) and is around 300 times sweeter than sucrose. Rebaudioside A is the next major steviol glycoside found in leaves (3.8%) and is around 450 times sweeter than sucrose [71]. Both have a bitterness and metallic aftertaste, but stevioside is reported to have increased off‐tastes compared to rebaudioside A. In solution, rebaudioside A is the most stable at between pH 4 and 8. Interestingly, recent research has focused on rebaudioside D and rebaudioside M as the most promising steviol glycosides due to decreased off‐tastes, yet these are present within the leaves at very low quantities of 0.2% and 0.1%, respectively. Rebaudioside M has been commercialised by PureCircle Limited (now a part of Ingredion) and the Coca‐Cola Company for food and beverage use [73]. Stevia was one of the earliest natural sweeteners to gain industrial interest and was recognised for use as a dietary supplement in the United States in 1994. It was, however, not approved for use in foods and beverages until 2008 when the FDA granted the designation to stevia extract (specifically Reb A) of being generally recognised as safe [74]. There have been several regulatory changes in the EU labelling of stevia extracts over the years as production methods have progressed, with a change from ‘steviol glycosides (E960)’ to ‘steviol glycosides from stevia (E960a)’ for those that have been extracted from the leaf and ‘enzymatically produced steviol glycosides (E960c)’ for those made through enzymatic purposes. Precision fermentation steviol glycosides were also approved for use in the EU in 2025, labelled as ‘steviol glycosides from fermentation (E960b)’.

#### 2.3.2. Monk Fruit

Monk fruit, also known as Luo Han Guo (the plant *Siraitia grosvenorii*), is an herbaceous perennial vine in the Cucurbitaceae (cucumber or melon) family. It is native to southern China and has been cultivated in the Chinese province of Guangxi for over 200 years [74]. Monk fruit′s sweetness is contributed by a group of terpene glycoside compounds called mogrosides. Five types of mogroside have been identified: siamenoside I, 11‐oxomogroside V, mogroside V, mogroside IVa and mogroside IVe, of which mogroside V is found in the highest concentration and is 100–300 times sweeter than sucrose [75, 76]. Monk fruit sweetener is highly soluble in water and heat stable, which facilitates its use in beverages and liquid formulations. Monk fruit exhibits a unique sensory profile that differs notably from sucrose. Specifically, it tends to produce a lower maximum sweetness intensity, along with lingering sweetness and the presence of off‐notes such as bitter, metallic and chemical [41, 42]. Strategies to enhance its taste performance have shown promise, particularly when monk fruit is used in moderate proportions (25% of a sweetener blend). Beyond this threshold, negative flavour attributes become more noticeable, especially bitter and metallic [77]. Monk fruit is extensively used in the Chinese medicine system, and in 2010, the FDA granted monk fruit sweetener as a safe food ingredient. As of October 2024, monk fruit decoctions have been declared as ‘not novel foods’ within both the United Kingdom and EU, thus increasing the ability to innovate in beverages with this natural sweetener, yet no maximum limits have been set.

#### 2.3.3. Sweet Proteins

Another class of intriguing natural sweeteners includes sweet‐tasting proteins isolated from different wild plants. Those discovered to date include thaumatin, brazzein, monellin, mabinlin, pentadin, curculin and lysozyme (from egg whites), as well as one sweet‐tasting modifying protein called miraculin. Unfortunately, many of these proteins are unstable at temperatures above 50°C making these difficult to implement in the food and beverage industry [43], yet thaumatin and brazzein are more heat stable. Thaumatins are found in the plant *Thaumatococcus daniellii,* native to West Africa, with the fruit called ‘katemfe’ or ‘miracle fruit/berry’. Thaumatin is comprised of six closely related proteins, which include thaumatin I, II, III, a, b and c. Thaumatin I and II are the main forms, yet all the proteins have a sweet‐tasting quality. Brazzein is found in the West African fruit ‘oubli’ from the plant *Pentadiplandra brazzeana* and is the smallest of the sweet‐tasting proteins. As these sweet proteins are found in relatively low amounts in the fruits available, research has been conducted to find alternative ways to produce them, with the most promising being through biotransformation and bioconversion. Thaumatin gene expression has been conducted in grains, fruits and vegetables, but the use of bacteria, fungi and yeast has been shown to give higher yields and faster growth [78]. Bioconversion has also been suggested as the most optimal way to manufacture brazzein, with corn used as a medium [43, 79]. Thaumatin is soluble in water and low levels of alcohol, stable at different pH levels and up to 120°C and elicits a sweet taste that has been estimated to be between 1600 and 3000 times sweeter than sucrose. It has been found to mask bitter and astringent notes found in pharmaceutical products, thus being used as a flavour modifier, yet it has also been found to have a cooling sensation and slight liquorice aftertaste [80]. It is normally used in combination with other sweeteners due to a delayed onset of sweetness and a long‐lasting lingering effect [37], and thus it is more commonly used as a sweetness enhancer (SE). Brazzein is around 2000 times sweeter than sucrose and the smallest of the sweet‐tasting proteins. It is stable at pH 2.5–8 and heat stable (up to 80°C), as well as water‐soluble. It has a shorter aftertaste than other lingering sweeteners on the market, can enhance flavour in beverages with citric acid and works well with stevia, Ace‐K and aspartame [44]. Currently thaumatin is the only sweet‐tasting protein that has been approved for use by the United States and EU. In terms of safety, there has been some discussion on the potential of sweet proteins being allergens due to their structure and sequence similarities to other allergens, but no direct correlation has been found [81]. Brazzein was recently approved for use by the FDA, meaning it can now be used as a sweetener in food and beverages in the United States.

#### 2.3.4. Rare Sugars

According to the International Society of Rare Sugars, rare sugars are ‘monosaccharides and their derivatives that are present in limited quantities in nature’ [82]. Out of over 50 naturally occurring rare sugars, D‐allulose, D‐tagatose, D‐sorbose and D‐allose have drawn the most scientific attention in terms of their physical, sensory and metabolic properties [70, 83]. D‐allulose (also known as D‐psicose) is an epimer of D‐fructose and is a promising sweetener due to its high solubility and antioxidant activity. D‐allulose delivers about 70% of the sweetness of sucrose whilst contributing significantly fewer calories (0.2 kcal/g) [70, 83]. Whilst its sweetness peaks lower than sucrose, its decline follows a similar trajectory. Notably, when blended with sucrose in equal parts, it replicates the sweetness curve of sucrose alone, offering potential as a partial sugar replacer in formulations [42]. D‐tagatose, a close structural relative to D‐fructose, is characterised by pleasant taste, bulking properties and is low in calories (2 kcal/g). It has a slightly faster sweetness rise rate than sucrose, closer to that of fructose. D‐tagatose offers about 92% of the sweetness of sucrose and mirrors the rapid onset profile of fructose. It maintains comparable sweetness intensity to sucrose across various concentrations typically found in foods (4.5%–18%). However, at 18% *w*/*v*, some participants detected a mild chemical off‐note [84]. D‐sorbose and D‐allose provide 70% and 80% of sucrose′s sweetness, but their caloric contributions remain unquantified in human studies [70, 83]. D‐sorbose reportedly has a taste profile similar to that of fructose, xylose, xylitol and glucose, with little research on the sweetness profile of D‐allose [70]. Amongst the rare sugars, only D‐allulose and D‐tagatose are generally recognised as safe by the FDA. The approval of D‐allulose in the EU is currently pending. D‐tagatose has been approved as a food in the United States, EU, Canada, Australia, New Zealand, Japan and South Korea.

#### 2.3.5. Sugar Alcohols

Sugar alcohols, also known as polyols, are low digestible carbohydrates that occur naturally in fruit, vegetables, mushrooms and algae. There are seven sugar alcohols that are defined as nutritive sweeteners according to EU legislation, which include sorbitol, mannitol, isomalt, maltitol, lactitol, xylitol and erythritol. These are obtained by substituting an aldehyde group with a hydroxyl one. The most interesting polyol to discuss in terms of use in beverages is erythritol, which unlike other polyols, is produced via enzymatic hydrolysis through yeast or lactic acid bacteria fermentation from wheat or corn starch. This is because the substrate erythrose is very expensive [85]. Erythritol is a four‐carbon sugar alcohol with a water solubility of 37% at 25°C and is stable at high temperature and pH as well as during light exposure [34]. It has a sweetening power of around 0.6–0.8 in comparison to sucrose, and thus it is used volume‐for‐volume and called ‘bulk sweetener’. This also means that it is often used in combination with other sweeteners [85]. It has also been described as having a mild cooling effect in the mouth and can improve mouthfeel and mask certain unwanted off‐flavours [37]. Sugar alcohols are not totally digested, and therefore excess ingestion can lead to gastrointestinal symptoms such as irritable bowel syndrome, abnormal flatulence and laxative side effects, especially for erythritol [47]. Products containing more than 10% of added polyols must therefore include the advisory statement ‘excessive consumption may produce laxative effects’. Recent research has shown that polyols have good antioxidant properties, reducing stress responses, as well as prebiotic properties that can contribute to healthy intestinal microbiota [46]. At this moment in time, erythritol is not approved for use in alcoholic drinks in the EU; however, it can be used at up to 1.6% in nonalcoholic flavoured beverages.

### 2.4. PAMs

Over the past decade, and since the identification of the sweet taste receptor, a new generation of SEs has been explored named PAMs, which work to enhance the sweetness of both artificial and natural sweeteners but have no sweet taste themselves [86]. This research was led by Senomyx Corporation (San Diego, CA, United States), which used high‐throughput screening and discovered a group of molecules that bind to the sweetness receptor without activating it whilst also causing sucrose to bind with a higher affinity. In their research, they found a group of SEs that were able to interact with sucralose and sucrose and enhance the intensity of their sweetness. For instance, SE‐1 and SE‐2 allowed a reduction of 50%–80% in the concentration of sucralose, with SE‐3 allowing a reduction of sucrose by 33% whilst also maintaining the sweetness intensity [86]. Other PAMs were also discovered by the same research group, namely, S2218, S6973 and S617, which are able to interact with sucrose, sucralose and fructose [87]. The mechanism for these SEs was shown in a later study to interact with the venus fly trap domain of T1R2. As discussed previously, sweeteners are able to bind near the hinge region of the venus fly trap domain and induce initial closure, yet for the SE molecules, they were found to bind near the opening of the pocket, strengthening the hydrophobic interactions between the two lobes and stabilising the closed conformation [53, 88]. In further studies, several unnatural tripeptides, named compounds 15, 17 and 19, were also shown to enhance the sweetness level of a 5% sucrose solution by 1.97 times the original level, although it is important to note here that this was only tested sensorially by three trained panellists [89]. Interestingly, it has been discussed that it is likely that PAMs such as those found in the study by Servant et al. [86] are not able to interact with larger sweetener compounds, such as stevioside and rebaudioside. These are made up of glucose moieties, which occupy the same space as sucrose/sucralose, and a steviol backbone, which overlaps with the same binding pocket that sweet enhancer molecules occupy, precluding their function [53].

PAMs have been approved for industrial use as flavour enhancers by EFSA, with the definition as ‘substances which enhance the existing taste and/or odour of a foodstuff’ [90]. They were also approved as a flavouring substance by the Flavour and Extract Manufacturers′ Association (FEMA). Although these PAMs are a step in the right direction for industrial sugar reduction, research is still needed to increase their effectiveness amongst different sweetener types and thus enable labelling of zero‐calorie products. In addition to this, the identification of PAMs from a natural origin would be beneficial as consumers seek more natural alternatives.

## 3. Temporal Profile Including Sweetness Onset and Lingering Sweetness

Of course, sweetness intensity is only one factor in the sensory properties delivered by sucrose. Sucrose is known to have an early sweetness onset and a quick sweetness decay, with onset described as the time it takes to reach the maximum sweetness sensation and lingering as the length of time of sweetness in the mouth. However, current models to predict and screen sweet‐tasting molecules do not take into account the complete sensory profile, including the dynamic sensory profile, and thus when sweeteners are used as a replacement, these do not match the same temporal profile that drives consumers′ acceptance and preference across the globe [91].

Over the years, there have been many ways to try to characterise the differences in sweetness onset and decay, with only one study to the authors′ knowledge using functional magnetic resonance imaging (fMRI) to investigate the brain response to the perception of sweet aftertaste. These researchers found that there was prolonged neural activation corresponding to the lingering aftertaste of aspartame, specifically in the primary taste cortex (insula) [92]. Other methods to measure this include the measurement of gustatory evoked potentials (GEPs), with one study showing that the GEPs were longer when stimulated by sweeteners such as aspartame and stevia in comparison to sucrose [93].

In sensory science, there are also multiple techniques to collect temporal data on the taste profiles of sweet‐tasting molecules. Many researchers have conducted studies using techniques such as TI, multiattribute time intensity (MATI), temporal dominance of sensations (TDS) and temporal check‐all‐that‐apply (TCATA) [42, 63, 91, 94–97]. Three recent studies have used TCATA to characterise and compare the dynamic properties of a selection of sweet‐tasting molecules, which included carbohydrate sweeteners and both artificial and natural sweeteners. Interestingly, and contradictory to other studies, these reported that nearly all sweeteners reached peak sweetness citation within approximately the same time frame as sucrose (~8–10 s) [42, 94, 96]. Yet, other researchers have found that Ace‐K had a faster onset of sweetness in comparison to sucrose [63, 64] and monk fruit had a delayed onset [41]. Andersen et al. [94] reported that these conflicting findings over many decades could be due to the different sweetness levels in the studies, with research showing that the lingering of aspartame only appeared at 9% sucrose equivalent concentrations and not at 5% [95]. In addition to this, a recent study published by Karl et al. [91] describes the taste and temporal properties of more than 30 sweeteners equivalent to 5% sucrose solutions. The authors concluded that the longer lasting lingering is associated with more complex, heavier molecules, such as stevioside, NHDC, thaumatin and neotame, indicated by the number of double bonds, ketones, aromatic rings and MlogP. For steviol glycosides in particular, the authors hypothesised that the undesired side‐tastes, as well as the onset and lingering of the sweet sensation, are based on the core structure of steviol glycosides rather than on the variable side chains. They also discussed that this lingering sweetness might be based on a higher affinity to the binding sites of the sweet receptor, with a delayed onset due to an inferior fit of a compound to the respective binding site [35, 42, 84, 91, 98]. Other researchers have looked at the idea that sweeteners may interact with salivary proteins, named mucins. It is hypothesised that these delay initial receptor binding by affecting small molecule diffusion rates through a hydrogel layer, with smaller polar molecules, such as sucrose, able to make the journey faster. In addition to this, they believed that amphipathic sweetener molecules engage in nonspecific binding to hydrophobic sites in this hydrogel layer, delaying the time it takes to reach the sweetener receptor. Furthermore, after disassociating from the receptor, they bind again to the hydrogel, enabling iterative receptor binding and increasing lingering sweetness [99, 100].

Solutions to counteract this difference in the temporal profile of sweeteners in comparison to their sucrose counterpart have been patented over many years. Tannic acid was used to reduce the lingering sweetness of sucralose in beverages [101], whilst a blend of both a high‐intensity sweetener, such as rebaudioside D or M, and a sweet lingering reduction compound, such as glucose, lactose, galactose, xylitol, thaumatin, brazzein and cyclamic acid, was suggested [102]. DuBois et al. [100] developed technologies to accelerate sweetener diffusion to and from sweetener receptors by using calcium salts. In their research, they found that the use of CaCl_2_, MgCl_2_ and KCl significantly reduced the sweetness linger of rebaudioside A when used at a concentration of 20 mM; however, at these concentrations, a significant off‐taste (bitter and salty) was also prevalent. Therefore, they decided to blend these salts to create a taste modulator composition and discovered that these worked synergistically to not only reduce sweetness linger but also enhance sweetness and mouthfeel intensity of numerous other artificial and natural sweeteners [100].

Although knowledge around the mechanism for sweetness onset and linger is increasing, only a few solutions have been published or patented in the search to move the temporal profile of sweeteners closer to their sucrose carbohydrate counterpart. To the authors′ knowledge, none of these solutions have shown a complete match in comparison to sucrose onset and decay, showing that there is still a way to go to improve both artificial and natural sweeteners. Other drawbacks discussed include the intensive resources needed to screen and reliably test potential sweetness modulators, with fMRI, EEG and sensory studies proving timely and costly [100].

## 4. Masking

The addition of sucrose can also help to mask the unwanted tastes found in products, including sour and bitter tastes. Many publications have looked at the interaction between two basic tastes to understand their synergism and antagonism effects [103–108]. In addition, researchers have looked into the effect of dose response rate and found that a low strength of sucrose had both enhanced and suppressed effects on different taste sensations, yet at medium or high strength concentrations, it suppresses other tastes [35, 103, 104, 108]. More recent studies have found that sour solutions (citric and tartaric acid) have a suppressive effect on the sweetness from sucrose [105, 107].

Interactions can also occur between noncarbohydrate sweeteners, used to replace sucrose, and the taste components within a product. Bonnans and Noble [109] investigated the interaction between aspartame and citric acid using a TI protocol. They found that sweeteners suppress the perception of sourness more than citric acid suppresses the perception of sweetness. This effect was found by comparing peak intensities in different concentrations of sweetener and citric acid. However, Wu et al. [110] investigated different levels of citric acid in a lemonade model matrix sweetened with either sucrose or steviol glycosides using TDS and TCATA. They found that citric acid did not affect the sweetness perception of steviol glycosides, whereas the sweetness of sucrose was suppressed.

In addition to this, many of the solutions suggested to replace sucrose have also been found to have bitter off‐tastes, and it has been found that both artificial and natural sweeteners (such as saccharin, Ace‐K and steviol glycosides) activate one or more bitter taste receptors named T2Rs [67, 111, 112]. This section will discuss the masking solutions for off‐tastes and flavours for both removal of sucrose and for addition of sweeteners.

### 4.1. Bitter Blockers

One mechanism used to help mask the off‐tastes caused by the removal of sucrose, or the addition of sweeteners, is the use of ‘bitter blockers’. Bitter blockers can be defined as compounds that modify bitter taste by interacting with the bitter taste perception pathway, interfering with taste receptors or the taste‐transduction mechanism [113]. Bitter tastants have been found to interact with TAS2R, with the activation of G‐protein gustducin upon stimulation, further raising intracellular calcium levels and activating the transient receptor potential cation channel M5 (TRPM5). This in turn generates action potential and the release of ATP, leading to the activation of taste perception in the brain. Bitter blockers have been found to work as antagonists by targeting hTAS2R bitter receptors, thus preventing the activation of gustducin and inhibiting taste perception [67, 114].

A comprehensive review conducted by Andrews et al. [113] listed studies that evaluated the efficacy of bitter blockers, with a focus on their use within the pharmaceutical industry. Unfortunately, only some of these have been tested with sweeteners as the agonist, with most being tested with typically bitter solutions such as quinine. However, one solution, with Ace‐K and saccharin as the agonists, included GIV3727 (4‐2,2,3‐trimethylcyclopentyl butanoic acid), a compound able to significantly reduce the bitter taste intensity in taste panels of both sweeteners from ‘moderate’ to ‘barely recognisable’ [114, 115]. Flavonoids, such as sakuranetin, 6‐methoxysakuranetin and jaceosidin, from *Eriodictyon californicum*, were also found by the same research group to inhibit the response to saccharin by more than 50% [116]; however, there are inconclusive results on the safety of these compounds at the doses required for bitter blocking [113]. *β*‐Cyclodextrin and homoeriodictyol sodium salt were also found to interact with sucrose and rebaudioside A to reduce the bitterness of (+)‐catechin without an impact on the flavour profile [117]. Salt solutions, such as zinc sulphate and magnesium sulphate, have been found to reduce perceived bitterness; however, zinc sulphate was found to also reduce sensory panellist perception of sweetness and increase astringency [118]. Other research looked at the ability of antagonists such as gamma‐aminobutyric acid (GABA) and N,N‐bis(carboxymethyl)‐l‐lysine (BCML) on bitter agonists such as quinine, discovering that although they act as bitter blockers, they can also deliver an umami taste [119]. Other bitter‐blocking compounds include flavan‐2‐ol‐spiro‐C‐glycosides reaction products and 1‐carboxymethyl‐5‐hydroxy‐2‐hydroxymethylpyridinium salt [120, 121].

It has previously been discussed that more research is needed on selective antagonists of the TAS2Rs, as most has only focused on nonselective [122]. In addition to this, the compatibility of information from different evaluation methods and the safety of these bitter masking compounds were also discussed as gaps in research [123]. Finally, the use of some of these bitter blockers in beverage solutions with sweeteners as the agonists is yet to be explored.

### 4.2. Blending Sweeteners and Production Processes

In the food industry, it is common to combine several low‐calorie and high‐potency sweeteners, because a single noncarbohydrate sweetener cannot fully replicate the sensory or physicochemical functions of sucrose. The use of sweetener mixtures can enhance the overall sweetness and sensory quality by suppressing the undesirable flavours inherent in high‐potency sweeteners [124]. This was discovered in the 1950s, with a blend of saccharin and cyclamate showing a reduction of bitterness [125]. Aspartame and Ace‐K blends were also found to work in synergy to enhance sweet taste [126], with blends of bulk and high‐potency sweeteners showing significantly lower intensities of bitter and liquorice flavour and aftertaste [127, 128]. Later, it was found that this was due to the sweeteners inhibiting each other′s bitter taste receptors, therefore minimising bitterness intensity [106, 129].

There are also certain production methods which have been developed to reduce the off‐tastes of certain noncarbohydrate sweeteners, which include coating or microencapsulation. These processes have been coveted as promising technology for both improving physical properties such as resistance to high temperatures and moisture and preserving structural integrity, as well as improving sensory properties, by decreasing aftertaste, bitterness and off‐flavours [130]. For instance, the use of spray drying as the microencapsulation method of steviol glycosides, with maltodextrin and inulin as the carrier, showed a significant reduction in bitter aftertaste [131, 132]. D’hoore et al. [133] also patented a combination of NHDC with *γ*‐cyclodextrin which reduced lingering aftertaste and onset time, as well as showed a synergistic effect by enhancing sweetness level.

### 4.3. FMPs

FMPs can be described as flavourings which are added to food to impart or modify the odour or taste. Some of these effects can be to change the temporal profile of a substance by altering the onset or duration of a particular characteristic or intensifying or reducing specific flavour characteristics (e.g., increase fruitiness or decrease metallic off‐notes). If these products modify only the sweetness, sourness or saltiness, these cannot be the primary effect. The effect of these FMPs can be evaluated using sensory testing over a two‐stage process. ‘Test 1’ uses a 2‐alternative forced choice (2‐AFC) test to demonstrate that the substance does not have an inherent sweetness. This is performed using a sample of sucrose at its recognition threshold concentration as the control, with the FMP test sample showing lower sweetness compared to the control sample to ensure it passes. It can then progress to ‘Test 2’, which uses either 2‐AFC or descriptive profiling to determine the effect of the FMP on other sensory attributes. In the EU, FMPs can be labelled as natural flavourings if they meet regulatory requirements.

One example of an FMP is the sweetener NHDC, which is an authorised flavouring substance under Regulation 1334/2008 at a level of up to 5 mg/kg. At low concentrations, it can increase specific attributes of a product, such as perceived fruitiness or a ‘jammy’ characteristic, whilst also reducing the perceived bitterness [134]. However, at higher concentrations, it can impart sweetness and thus can only be used as a sweetener. It has also been combined with beta‐ and gamma‐cyclodextrin to work as a taste‐masking agent against the bitter, astringent and metallic off‐tastes [135]. Unsurprisingly, there is little published data on the compounds used as FMPs in the food industry due to intellectual property rights; however, it has been discovered that some steviol glycosides, when used below the sweetness detection level, have modifying properties. The product make‐up includes specified ratios of different steviol glycosides (Reb A through to Reb M), with many of these patented and protected by individual suppliers.

Finally, certain flavours have been found to mask the unwanted characteristics of different sweeteners. Mielby et al. [136] discovered that lime flavour can mask the aftertaste of stevia in fruit beverages, with further research by Tong et al. [137] evaluating the effect of key aroma compounds from limes on the sensory properties of rebaudioside A using descriptive analysis and TI sensory techniques, as well as molecular docking. The authors found that compounds such as D‐limonene, citral and *γ*‐terpinene can exhibit sweet‐enhancing effects, as well as mask off‐flavours such as bitter, metallic, astringent and liquorice and also alter the consumption experience by extending sweetness and bitterness duration [137].

## 5. Flavour Intensity and Delivery

The interactions between sucrose and flavour components in beverages and their effect on sensory properties have been a topic of debate over many years, with conflicting results from different studies. Yet, there is no doubt that the addition of sucrose can alter flavour intensity and delivery within a product. The idea of a ‘salting out’ effect was first suggested by Friel et al. [24], which was described as the effect of increasing sucrose concentration on the release of specific flavour compounds into the headspace, due to an interaction between sucrose and water [24, 25, 138]. In addition to this, other researchers found that the release rate of more volatile compounds intensified by increasing sucrose content, thus causing a significant shift in the flavour profile [25, 139]. Nahon et al. [139] showed that an increase (from 0% to 60% *w*/*v*) in the sucrose concentration in a solution leads to an increased release of the flavour compounds with short gas chromatography/flame ionisation detection (GC/FID) retention times and a decreased release of the compounds with longer retention times. In contrast, Hewson et al. [140] found that the addition of sugars (namely, glucose and fructose at 68 and 150 g/L) resulted in an overall decrease in volatiles in the headspace when compared to volatiles‐in‐water samples; however, the citrus flavour intensity was found to significantly increase by a trained sensory panel. They concluded that the flavour enhancement of the beverages was not fully explained by physicochemical interactions within the beverage matrix and instead were due to multimodal taste interactions.

### 5.1. Flavours to Enhance Sweetness

With this in mind, it is interesting to explore multimodal taste interactions with the addition of appropriate aromas to enhance sweet intensity. Multimodal interactions occur due to learning and association through previous experiences with taste and aroma combinations [28]. Previous studies report that flavours such as vanilla, caramel and fruits are directly related to the increased perception of sweet taste [141]. For instance, Velázquez et al. [142] evaluated cross‐modal interactions in vanilla milk desserts and suggest that this strategy can minimise the sensory changes caused by sugar reduction. Schwieterman et al. [143] evaluated fresh strawberries and identified more than 20 volatile compounds capable of increasing the perception of sweetness, independent of the glucose, fructose or sucrose content. Studying the relationship between flavour and taste in this way may contribute to the development of products with reduced sugar content, without compromising the acceptance of these foods.

#### 5.2. Flavour Enhancers

To tackle this in the food industry, flavour enhancers have often been used. As discussed in section 2.4, PAMs are one example of these substances. The most well‐known flavour enhancer is monosodium glutamate (MSG), which is often used in savoury applications to enhance umami taste. There has been little published data on flavour enhancers for sweet applications, yet the sugar alcohol erythritol has been found to improve the flavour profile of energy‐reduced beverages and has been approved for use in the EU as a flavour enhancer at low concentrations (1.6%) [144, 145]. Maltol is also another commonly used flavour enhancer, with a cotton candy aroma and fruity note at lower concentrations; therefore, it is able to help enhance the sweetness [146]. Ethyl maltol has also been patented for use as a flavour enhancer [147]. One problem with using such volatile compounds in enhancing sweet taste is that their own characteristic caramel‐like aromas may be inappropriate for the food products targeted for enhancement [148]. Again, there are few published articles on potential novel flavour enhancers, and thus more research needs to be conducted in this area to tackle flavour intensity and delivery challenges found when reducing sugar.

## 6. Mouthfeel

Finally, sucrose provides viscosity, texture and mouthfeel in beverages through various physicochemical interactions with water and other solution components [26]. Thus, consumers frequently describe sugar‐free beverages as ‘thin’ or ‘watery’, and for many, these sensations are perceived as negative.

The primary mechanism by which sucrose increases viscosity is through its interaction with water molecules. Sucrose is highly soluble and interacts extensively with water through hydrogen bonding due to its multiple hydroxyl (–OH) groups. Each sucrose molecule can form hydrogen bonds with surrounding water molecules, reducing the mobility of water molecules and leading to a decrease in free water, which consequently increases solution viscosity [149, 150]. The concentration of sucrose in a solution has a nonlinear effect on viscosity. At low concentrations (< 40%), sucrose minimally increases viscosity due to the ample presence of free water, which allows fluidity. However, as sucrose concentration increases (above 50%–60% weight/volume), the sucrose molecules begin to form a dense network, increasing solution viscosity significantly and giving the solution a syrupy texture [27]. This concentration‐dependent effect is well documented, showing a sharp viscosity increase as sucrose approaches saturation levels. Furthermore, as the sucrose concentration approaches saturation, the solution′s viscosity rises due to the crowding of sucrose molecules, which restricts water movement even further.

Replacing lost body/mouthfeel is not as straightforward as might be imagined; however, the seemingly simple solution of adding back bulk in the form of ingredients such as maltodextrins clearly is counterproductive due to it being calorific [151]. One suggested solution to this problem is through the use of hydrocolloids, which have similar water‐binding properties compared to sucrose. These polymers form hydrated networks that can create the perception of thickness and creaminess in beverages. The most significant hydrocolloids in terms of market value are starches and modified starches, followed by gelatin. A number of nonstarch hydrocolloids (‘gums’) are of increasing commercial value (e.g., xanthan, guar and carrageenans) [152]. Gallardo‐Escamilla et al. [153] indicated that high‐methoxyl pectin (HMP), carboxymethyl cellulose (CMC) and propylene glycol alginate (PGA) can be used to significantly increase the instrumental and perceived (sensory) viscosity of liquid whey to match the physical viscosity of lactic beverages. Another solution is using sugar alcohols, like sorbitol and erythritol, which can mimic sucrose′s texture to a certain extent. Although their main role is as sweeteners, they can also contribute to mouthfeel enhancement due to their osmotic and bulking properties. de Cock and Bechert [144] demonstrated that erythritol can improve taste quality by adding body and mouthfeel to functional beverages. Fibre syrups, such as inulin, can also be used as a bulking agent in a sugar reduction reformulation strategy. In a recent study, it was shown that inulin contributed to the physiochemical characteristics of cagaita (South American fruit) beverages (with increased total soluble solids) [154]. Finally, mineral salts have also been found to boost sugar‐like mouthfeel. In a recent publication by DuBois et al. [100], there was a dramatic increase in sugar‐like mouthfeel observed in formulations with K+, Mg_2_+ and Ca_2_+ mineral salt compositions. The authors speculated that this improvement may be driven by the activation of taste bud cells expressing the calcium‐sensing receptor by both Ca_2_+ and Mg_2_+ ions, with possible allosteric modulation by K+ ions. As has also been discussed in previous sections on masking, FMPs can be used to enhance the mouthfeel of products. However, none of these solutions deliver the exact same mouthfeel as sucrose, and one should also bear in mind the interactions between these compounds and other ingredients.

## 7. Future Perspectives

### 7.1. Conflicting Consumer Perspectives

Regarding healthy and natural sweeteners, consumers have every right to be confused about making positive choices in their daily routine, with recent WHO reports on the possible carcinogenicity of long‐term exposure to artificial sweeteners, and suggestions that noncarbohydrate sweeteners should not be used for weight control or reducing the risk of noncommunicable diseases. Thus, a major barrier to promoting the adoption of noncarbohydrate sweeteners is addressing consumer perceptions and misconceptions. For some, known store cupboard ingredients on the back of pack are the only option, and with the introduction of new novel natural sweeteners named as steviol glycosides, allulose and brazzein, consumer scepticism persists. This stems from unfamiliarity with these sweeteners, their taste profiles and lingering concerns about their safety and efficacy compared to their more well‐known artificial counterparts. Therefore, future efforts should focus on holistic consumer education on the benefits of switching from sucrose to noncarbohydrate sweeteners, with a focus on the benefits of natural solutions. Tailoring educational campaigns through interactive tools, such as taste trials or digital content comparing these sweeteners with traditional sugars, could foster familiarity and trust.

Another aspect to consider for the future is that consumers report that they are looking for more environmentally sustainable solutions. One of the aspects which seems to be underreported by food and drink organisations is the potential for both artificial and natural sweeteners to be more environmentally sustainable compared to their sucrose counterpart. Lifecycle assessments have been reported for different sweeteners, with many reporting that these noncarbohydrate sweeteners use less land footprint and resources compared to high fructose corn syrup, sugar from sugarcane and sugarbeet [40, 155]. On the other hand, it is now being reported that some artificial sweeteners, such as saccharin and sucralose, are accumulating in the environment because they are excreted from the human body unchanged. Research shows conflicting results on the effect this will have on both aquatic and land environments, but it will be interesting to see what the future holds in this area.

### 7.2. Regulatory Barriers

Another key aspect which means that innovation in the sugar‐free and sugar‐reduced world cannot progress at a more rapid pace is the tedious and cumbersome processes of regulatory approval for the use of novel ingredients (such as sweet proteins) or new technologies (such as biofermentation) in the food industry. Although these practices are extremely important in terms of protecting the safety of the consumer, they can limit creativity and innovation to some extent. For instance, although steviol glycosides have been approved for use in some beverage categories within the EU (including energy drinks and nonalcoholic beers), there are still other categories for which they are awaiting approval. Unfortunately, many novel natural sweeteners have not been approved for use in alcoholic beverages in the EU, meaning that options are very limited for breweries and distillers in the development processes.

### 7.3. Advancement of Scientific Research

As discussed earlier in this review, there are many sensory challenges linked with noncarbohydrate sweeteners, ranging from delayed sweetness onset and lingering aftertaste to off‐flavours that need to be masked. Researchers have developed technologies to try to tackle these challenges, yet there is still no perfect solution. The flavour industry is now looking into the use of receptor‐based discovery of not only sweet, but also bitter, salty and cooling sensations, with rapid screening of natural ingredients and progressed understanding of the mechanisms of molecules binding to different receptors. Unfortunately, however, much of the knowledge collected is treated as intellectual property, and thus the field cannot progress without multiple industries collaborating to reach a viable solution.

In addition to this, the use of more advanced techniques, such as fMRI has been used to investigate the differences in brain activation between sucrose and artificial sweeteners, showing that both activated the primary taste pathway, but only sucrose and glucose activated the reward‐related areas of the brain [156, 157]. A recent review discussed advances in this area [158]; however, there are still unanswered questions in terms of the brain response to natural sweeteners, such as rare sugars, as well as the temporal profile and lingering effect.

Numerous studies have also used aqueous solutions as a model matrix, yet in complex solutions, it appears that sweeteners move closer to their sucrose counterpart due to taste–taste, taste–flavour and taste–texture interactions [94]. To the authors′ knowledge, no research has delved deeper into understanding the key characteristics driving these differences amongst matrices, using techniques such as design of experiments, with factors such as acidity, pH, sweetness level and ingredient build‐up, which could significantly improve developments in this area.

### 7.4. Final Remarks

Reduction or removal of sucrose is not a simple task, with no ideal single solution to this challenge, indicating that there is something missing in this sector. This review has taken a holistic view of the product development process by discussing each sensory challenge that occurs when reducing sugar and suggested recommendations to improve the overall product. For the industry to move a sugar‐free product closer to its full‐sugar counterpart, however, cross‐functional teams are needed to understand the effect the replacement can have on brain chemistry, health, dietary choices, sensory properties, stability, shelf‐life, environmental factors and consumer preference. Yet, we may also need to find a solution to tackling this problem in a different way.

**Nomenclature**
ADI: acceptable daily intake
2‐AFC: 2‐alternative forced choice
EFSA: European Food Safety Authority
FDA: the United States Food and Drug Administration
FEMA: Flavour and Extract Manufacturers′ Association
FMPs: flavours with modifying properties
GPCRs: G protein‐coupled receptors
JECFA: Joint FAO/WHO Expert Committee on Food Additives
MATI: multiattribute time intensity
NHDC: neohesperidin DC
PAMs: positive allosteric modulators
TCATA: temporal check‐all‐that‐apply
TDS: temporal dominance of sensations
TI: time intensity
UNESDA: Union of European Soft Drinks Associations

## Conflicts of Interest

The authors declare no conflicts of interest.

## Author Contributions

**Imogen Ramsey:** conceptualisation, writing – original draft, writing – review and editing, project administration. **Jing Liu:** conceptualisation, writing – original draft, writing – review and editing. **Jan Hendrik Swiegers:** writing – review and editing. **Olayide Oladokun:** writing – review and editing.

## Funding

No funding was received for this manuscript.

## Data Availability

Data sharing is not applicable to this article as no datasets were generated or analysed during the current study.
